# Abnormal Default Mode Network Homogeneity in Treatment-Naive Patients With First-Episode Depression

**DOI:** 10.3389/fpsyt.2018.00697

**Published:** 2018-12-18

**Authors:** Yujun Gao, Menglin Wang, RenQiang Yu, Yaping Li, Ying Yang, Xiangxiang Cui, Jinou Zheng

**Affiliations:** ^1^Department of Neurology, The First Affiliated Hospital, Guangxi Medical University, Nanning, China; ^2^Department of Otorhinolaryngology and Head and Neck Surgery, The First Affiliated Hospital, Guangxi Medical University, Nanning, China; ^3^Department of Radiology, The First Affiliated Hospital, Chongqing Medical University, Chongqing, China; ^4^The First Affiliated Hospital, Guangxi Medical University, Nanning, China

**Keywords:** depression, default mode network, network homogeneity, attentional network test, rest-fMRI

## Abstract

**Background and Objective:** The default mode network (DMN) may be an important component involved in the broad-scale cognitive problems seen in patients with first-episode treatment-naive depression. Nevertheless, information is scarce regarding the changes in network homogeneity (NH) found in the DMN of these patients. Therefore, in this study, we explored the NH of the DMN in patients with first-episode treatment-naive depression.

**Methods:** The study included 66 patients and 74 control participants matched by age, gender, educational level and health status who underwent resting-state functional magnetic resonance imaging (rs-fMRI) and the attentional network test (ANT). To assess data, the study utilizes NH and independent component analysis (ICA). Additionally, Spearman's rank correlation analysis is performed among significantly abnormal NH in depression patients and clinical measurements and executive control reaction time (ECRT).

**Results:** In comparison with the control group, patients with first-episode treatment-naive depression showed lower NH in the bilateral angular gyrus (AG), as well as increased NH in the bilateral precuneus (PCu) and posterior cingulate cortex (PCC). Likewise, patients with first-episode treatment-naive depression had longer ECRT. No significant relation was found between abnormal NH values and the measured clinical variables.

**Conclusions:** Our results suggest patients with first-episode treatment-naive depression have abnormal NH values in the DMN. This highlights the significance of DMN in the pathophysiology of cognitive problems in depression. Our study also found alterations in executive functions in patients with first-episode treatment-naive depression.

## Introduction

Depression is a frequent complex disorder of unclear pathogenesis which is typically characterized by persistent feelings of sadness, loss of interest, reduced energy, a pervasive loss of pleasure, cognitive impairment, and vegetative symptoms (1, 2). Depression is currently known to affect a large number of people globally (http://www.who.int/zh/news-room/fact-sheets/detail/depression), being one of the main causes for disability worldwide and having a substantial socio-economic burden (3). Currently available treatments for depression are far from ideal due to high recurrence rates along with frequent and intolerable side-effects (4).

Increasing evidence has shown depression may be regarded as a disorder of neural networks (5, 6). In particular, the default mode network (DMN) has received growing attention. Previous research has found that the DMN is significantly involved in the neurobiology of depression and has been proposed as a biomarker for treatment response, as DMN activity appears to predict levels of depressive rumination (2, 7–9). The DMN comprises several structures: the medial prefrontal cortex (MPFC), the precuneus (PCu), the anterior/posterior cingulate cortex (A/PCC), and the medial, lateral and inferior parietal cortex (10–13). Recently, the lateral temporal gyrus and the cerebellar crus 1 and 2 have been found to participate in DMN connectivity(13, 14). DMN activity is usually higher at rest and decreases during task-related cognitive processes (15). The DMN has been correlated with monitoring external environments, keeping self-consciousness, producing spontaneous thinking, self-related feelings, memory, the process of cognition, negative ruminations, complex self-referential stimuli, and some special mind-states (16–20).

Previous investigation on depression has demonstrated abnormal resting state connectivity in the DMN; however, findings remain inconsistent on whether connectivity is increased (21–23), decreased (24, 25) or even both (26, 27). Recently, a study reported no correlation between the MPFC and the PCC (28). These discrepancies may be due to several aspects. Furthermore, differences in methodology and limited sample sizes can significantly affect results. Factors such as drugs, treatment methods, illness duration and severity can also contribute to DMN abnormalities. For example, antidepressants can reduce functional connectivity in the DMN (29, 30), while both electroconvulsive therapy and transcranial magnetic stimulation have been observed to change functional connectivity in the DMN (31, 32). Hence, studies on first-episode treatment-naive subjects with depression may have the advantage of lessening confounders.

Network homogeneity (NH) has been widely studied in patients with attention-deficit/hyperactivity disorder, somatization, schizophrenia, and their unaffected siblings (6, 33–40). This method studies a given network without specifying the requirement of localization of network abnormalities. Thus, it assesses the homogeneity of a whole network, an aspect of intrinsic network organization that has long been overlooked. As a voxel-wise measurement approach, NH correlates with all other voxels in a provided interest network. For a given voxel, its average correlation is regarded as an NH value. Homogeneity is defined as the time series comparability for a provided voxel or others in a given network. DMN is associated with cognitive functioning, especially executive function. When the brain is performing tasks, the DMN is negatively activated. Based on studies of DMN abnormalities in patients with depression (40), we hypothesizes that patients with first-episode treatment-naive depression show abnormal DMN homogeneity, which may be related to clinical variables such as illness severity and executive control reaction time (ECRT).

## Materials and Approaches

### Ethics Statement

All subjects signed the written informed consent before participating in this investigation. The study was approved by the ethics committee of the First Affiliated Hospital, Guangxi Medical University and in accordance with the Declaration of Helsinki.

### Subjects

This study included 66 patients with first-episode treatment-naive depression and 74 control participants, all of them recruited from the Department of Neurology, Psychology and Radiology of the First Affiliated Hospital of Guangxi Medical University. Patients were diagnosed following criteria from the Diagnostic and Statistical Manual of Mental Disorders, fourth edition (DSM-IV), by independent assessments from two psychiatrists. We used the 17-item Hamilton Rating Scale for Depression (HRSD-17) to evaluate depression severity. All patients had total scores ≥17 in the HRSD-17 on the day of MRI evaluation. The exclusion criteria were: left-handedness; family history of neurological disorders, severe physical illnesses and substance abuse; pregnancy; findings of abnormal cerebral structures after the initial MRI scanning, and the presence of other psychiatric disorders, such as personality disorders or schizophrenia and related disorders. In total, 74 individuals were included in the control group, matched for age, gender, educational status and overall health, and it is noteworthy that exclusion standards are the same for depression patients.

### Behavioral Paradigm

The attentional network test (ANT) was designed by Fan et al. (41), which was presented using Eprime and E-Studio software (Psychological Software Tools, Pittsburgh, PA, USA). The standard procedures for ANT were followed (https://www.sacklerinstitute.org/cornell/assays_and_tools/ant/jin.fan/). In the central testing screen, a “+” sign was placed and regarded as the fixation point. A stimulus signal could be generated above or below the central screen in the form of a target → or a foil ^*^. Four situations involved foils: No foil, one foil in the central part, one foil above the central screen and another one below it, and one foil either above or below the central screen. Arrows could appear in the following ways: A single arrow, five arrows in a direction, and five arrows in different directions. Subjects were required to assure target orientation correctly and quickly. ECRT was calculated by subtracting the consistent arrow direction reaction time (RT) from the inconsistent arrow direction RT. Longer ECRT represents lower efficiency of the executive control network.

### Resting-State Functional Magnetic Resonance Imaging

An Achieva 3TMRI scanner (Philips, Netherlands) was utilized for resting-state functional magnetic resonance imaging (rs-fMRI). Patients were asked to lie down and close their eyes but remain awake. A prototype quadrature birdcage head coil filled with foam was used to minimize head movement. Functional imaging had the following parameters: ratio of repetition time to echo time (TR/TE) (2,000/30 ms), slice thickness (5 mm), pitch (1 mm), field of view (240 × 240 mm) and flip angle (90°). On the structural scan (T1-weighted), the following settings were used: spin-echo sequence, repetition time (TR) = 20 ms, echo time (TE) = 3.5 ms, slice thickness = 1 mm, and field of view (FOV) = 24 × 24 cm.

### Data Preprocessing

Imaging data from the rs-fMRI was preconditioned using the data processing assistant for resting-state fMRI (DPARSF) software (42) in Matlab. The first 10 time points were removed, and slice time and head motion were rectified to adjust the time series of images so that the brain is in the same position in every image (43, 44). No participants had more than 2 mm of maximal displacement in the x, y, or z axes and more than 2° of maximal rotation. The structure of each patient was registered to its functional image. The structure of each patient was divided, and a template was created to normalize the structures of the patients after they were defined according to the Montreal Neurological Institute (MNI) standard template, the standardization process of the spatial deformation of the modulation and the structure of the voxel size using 1 × 1 × 1 mm. Finally, the use of the structure of each patient to the function of the conversion matrix was also standardized to the MNI space. During the process of functional image normalization, head motion parameters, white matter signal, and cerebrospinal fluid signal were used as removal covariates (Nuisance regression), and voxel size of 3 × 3 × 3 mm was used as functional covariate. The obtained images were subsequently smoothed with an 8 mm full width at half-maximum Gaussian kernel, band pass filtered (0.01–0.1 Hz), and linearly detrended to lessen the effect of low-frequency drifts and physiologic high frequency noise. Several spurious covariates were removed, including a signal from a region centered in the white matter, 6 head motion parameters obtained by rigid body correction, and a signal from a ventricular ROI. The global signal removal may introduce artifacts into the data and distort resting-state connectivity patterns. Furthermore, the regression of the global signal may significantly distort results when studying clinical populations. Therefore, the global signal was preserved (45, 46).

### Default Mode Network Identification

Independent component analysis (ICA) was performed using the Group ICA utility to remove DMN components in templates from the GIFT fMRI toolbox (http://mialab.mrn.org/software/#gica) (46). Three procedures from the GIFT toolbox were utilized for ICA analysis: data reduction, separation of independent components and back rebuilding. On the consideration of every component, the voxel-wise one-sample *t*-test set a statistical map and a threshold. Based on Gaussian random field (GRF) theory, *p* < 0.01 represents a significant statistical modification of multiple comparisons. Voxel significance meets requirements at values of *p* < 0.01, and cluster significance values for *p* < 0.01. The study created masks for the parts included in the DMN. Finally, after combination, the DMN masks were utilized in the NH analysis.

### Network Homogeneity Analysis

The results of NH analysis were computed through the application of an in-house script in Matlab (33, 34). The DMN masks showed correlation coefficients between a provided voxel and all others. There is a definition of the correlation coefficient in average as the homogeneity of the provided voxel. Then, the averaged correlation coefficients were converted into z values through z-transformation, promoting normal distribution. The resultant values generated the NH map that finally underwent z-transformation for group comparison.

### Statistical Analysis

The study computed demographic information such as age, gender, and education degree, as well as imaging data from the patient and control groups. The two-sample *t*-test was applied for the comparison of continuous variables, while the chi-square test was employed to compare categorical data by using the IBM SPSS Statistics 22.0 software. With the purpose of measuring the discrepancies in the NH regional group, the two-sample *t*-test assisted the individual-level NH map into one group-level voxel wise *t*-test analysis. Later, in the DMN mask, through voxel-wise cross-subject statistics, the two-sample *t*-test was employed to analyze the NH maps. GRF theory is applied into the modification of significance level (*p* < 0.01) for multiple comparisons. (GRF corrected, voxel significance*: P* < 0.001; cluster significance*: P* < 0.01).

### Correlation Analysis

NH values are withdrawn from abnormal values in brain regions. After the evaluation of data normality, Pearson correlations can be found among the variables with *p* < 0.05 in statistics using the IBM SPSS Statistics 22.0 software.

## Results

### Subjects' Demographics and Clinical Features

Demographic information of the study participants is presented in Table 1. There were no significant discrepancies among the three groups regarding gender, age, and education years. ECRT was longer in the patient group.

**Table 1:** Characteristics of the participants.

**Demographic data**	**patients(*n* = 66)**	**NC(*n* = 74)**	**T(orx^**2**^)**	***P* value**
Gender(male/female)	66(30/36)	74(40/34)	0.16	0.45^a^
Age(years)	28.44 ± 7.6611	28.88 ± 6.67	0.36	0.57^b^
Years of education(years)	36 ± 2.40	12.98 ± 2.49	3.91	0.37^b^
HRSD score	25.88 ± 5.26	−	–	–
ECRT	153.13 ± 71.27	87.09 ± 29.78	7.29	0.00^b^

a *The p value for gender distribution was obtained by chi-square test*.

b *The p value were obtained by two sample t-tests*.

*NC, normal control; HRSD, Hamilton Rating Scale for Depression; ECRT, executive control reaction time*.

### DMN Maps as Ascertained by Group ICA

**Figure 1:**
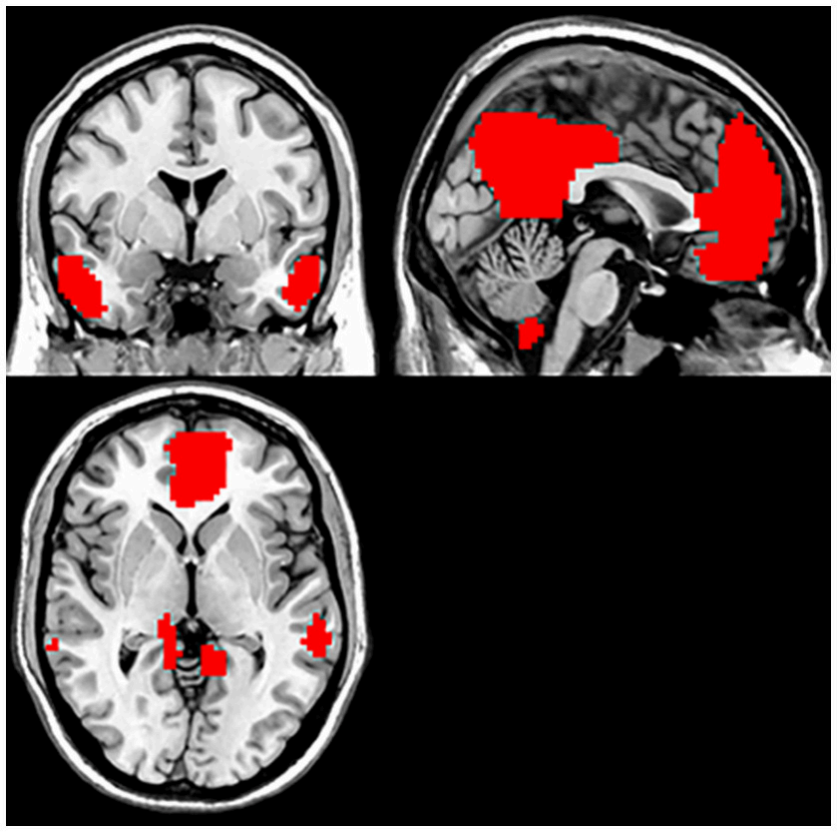
Default mode network (based on group-ICA with threshold at z≥5).

By employing ICA, DMN masks were removed from the control group. The parts involved in the DMN included the bilateral PCC/PCu, MPFC, ventral anterior cingulate cortex (ACC), lateral temporal cortex, parietal lobes (medial, lateral, and inferior), and cerebellum Crus 1 and Crus 2 (Figure 1).

### Group Differences in DMN Regarding NH

**Figure 2:**
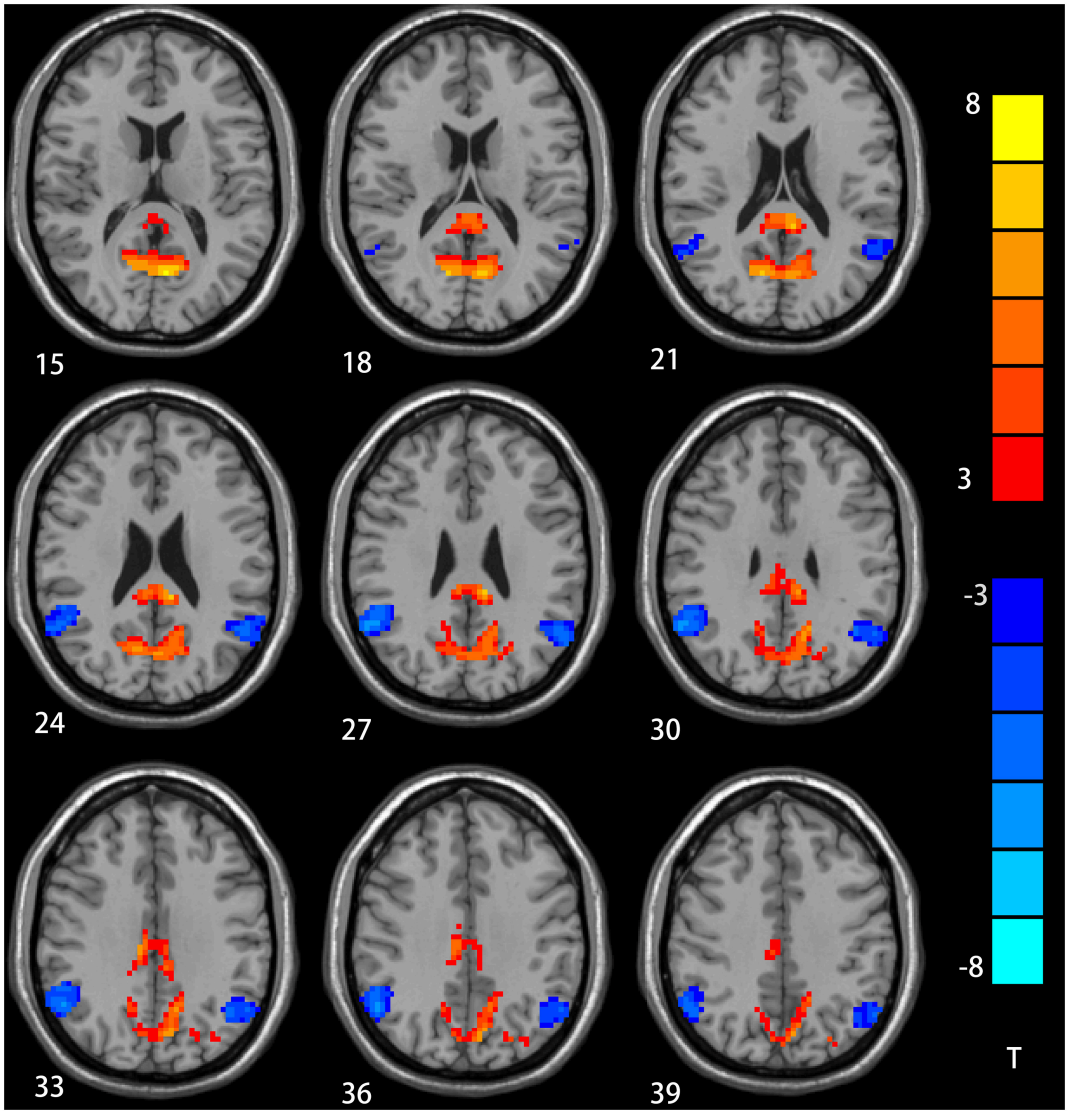
NH differences in the bilateral angular gyrus, enhanced NH in the bilateral precuneus (PCu) and bilateral postal cingulum cortex (PCC) between depression group and control group in statistical maps (Blue denotes lower NH and red denotes higher NH. Meanwhile, color bars point T values obtained from two-sample *t*-test. NH is the abbreviation of network homogeneity).

The two-sample *t*-test showed significant group discrepancies of NH values between patients and controls within the DMN masks. In comparison with the controls, patients with depression had lower NH in the bilateral AG and significantly higher NH in the bilateral PCC and PCu (Table 2 and Figure 2).

**Table 2:** Signification differences in NH values between the groups.

**Cluster location**	**Peak**	**(MNI)**		**Number of voxels**	***T* value**
	**X**	**Y**	**Z**		
**PATIENTS>CONTROLS**					
bilateral PCu	±6	−66	12	609	8.77
Bilateral PCC	±9	−36	27	207	7.45
**PATIENTS < CONTROLS**					
bilateral AG	±57	−54	27	224	−6.36

*MNI, Montreal Neurological Institute; PCu, Precuneus; PCC, posterior cingulate cortex; AG, angular gyrus*.

### Correlation of NH With Clinical Variables

Significant group discrepancies were found in the six regions where the averaged NH values were withdrawn (bilateral AG, bilateral PCC, and PCu). In the patient group, Pearson linear correlation analysis was implemented to explore the correlations among NH, ECRT, and illness severity. The results showed no significant correlations of NH with those clinical variables.

## Discussion

In this paper, NH was studied on the DMN of patients with first-episode treatment-naive depression. The latter showed significantly lower NH in the bilateral AG and significantly higher NH in the bilateral PCC and PCu compared to controls. These patients also had longer ECRT. However, no significant correlations were found among NH and illness severity or ECRT in any of the regions.

The cingulate gyrus is an important area where the frontal cortex, insula, amygdala, and hypothalamus interconnect. As a part of the limbic system, it participates in emotion regulation, cognitive function and self-control. PCC represents a core hub for the DMN and plays a key role in integrating self-relative information, retrieval of episodic memory, and autobiographical search. As reported by Maddock (47), unpleasant words can cause significant activation of PCC by task state-fMRI. On the other hand, Hagmann et al. (48) found that PCC can integrate information across the cerebral cortex through a graph theoretic analysis method. According to Marchetti et al. (49), lower fractional anisotropy (FA) values are found in PCC in major depressive disorder. PCC volume is significantly reduced in patients with depression (50, 51). The average metabolic rate of PCC appears to be increased in patients with depression, and can be reduced significantly by antidepressant treatment (52). In addition, a meta-analysis reported the PCC is reliably involved in autobiographical memory, prospection, navigation and theory of mind (53). On the other hand, many studies have observed that functional connectivity decreases in PCC in patients suffering with depression (21–24). One possible interpretation for this paradox is that there is a possible compensatory mechanism. However, some hold that increased PCC is an intrinsic characteristic of depression (54). Our findings suggest the increased NH in the bilateral PCC could contribute to depression.

The PCu—situated in the posterior DMN—intervenes in memory and processing of self-references, while its deactivation has been related to consciousness (55). Abnormal PCu activity can enhance self-references, which favors sleep disorders (56). In this study, we found that increased NH in bilateral PCu demonstrates decreased interaction with the DMN. At least two studies have found that abnormal PCu activity may be related to the genetic risk of depression (57, 58). Therefore, we surmise that increased NH values in the bilateral PCu might be associated with depression.

The AG, in the posterior of the inferior parietal lobe, is regarded as a main hub for various subsystems (59). The AG is involved in handling semantics, reading and comprehending words, dealing with numbers, retrieving memory, attention and spatial cognition, social cognition and inference (60). Mulders et al. (32) observed DMN coherence is significantly decreased in the AG in patients with depression. Similarly, Chen et al. found decreased connectivity in the AG within DMN inpatients suffering with first-episode, treatment-naive major depressive disorder, which is associated with higher autobiographical memory scores (61). Patients with depression are thought to have slower thinking and memory loss, which is associated with decreased NH values in bilateral AG.

It is universally acknowledged that the DMN is associated with executive functions, presenting increased activity at rest and decreased activity during the execution of oriented cognitive tasks (62, 63). Therefore, depression patients usually display functional executive impairment and longer ECRT. This parameter is measured by the ANT designed by Fan et al. (41). This test has been applied in the research of other conditions such as Parkinson's disease (64). Longer ECRT represents lower efficiency of the prompt executive control network. Damaged PCC/PCu can affect frontal lobe activity and disrupt execution functions (65). Based on these speculations, abnormal NH, ECRT and illness severity are assumed to be correlated. However, we found no correlations among these factors. This may be because the abnormal NH values in the DMN belong to a characteristic variety for those patients who are not limited by these factors or alternatively are due to the sample size.

Our study had a few limitations. The influence of physiological noises such as cardiac and respiratory rhythm cannot be completely removed. The sample size was notably restricted. Finally, our study focused only on alterations in the DMN, possibly neglecting significant changes in other brain regions.

Despite these limitations, our study results corroborate the importance of DMN in the pathophysiology of depression by highlighting the presence of abnormal NH values in the DMN of patients with first-episode treatment-naive depression. In addition, we have posited a method for the assessment of NH, which may improve the comprehension of the pathophysiology of depression in future studies.

## Author Contributions

YG designed experiment and wrote this article. MW, RY, YL, YY, and XC collected and analyzed these data. JZ guided the experiment and revised the article.

### Conflict of Interest Statement

The authors declare that the research was conducted in the absence of any commercial or financial relationships that could be construed as a potential conflict of interest.
